# Effect of the Morphology of Tungsten Oxide Embedded in Sodium Alginate/Polyvinylpyrrolidone Composite Beads on the Photocatalytic Degradation of Methylene Blue Dye Solution

**DOI:** 10.3390/ma13081905

**Published:** 2020-04-17

**Authors:** Eman M. Elsayed, Mohamed S. Elnouby, M. H. Gouda, Noha A. Elessawy, D. M. F. Santos

**Affiliations:** 1 Fabrication Technology Department, Advanced Technology and New Materials Research Institute, City of Scientific Research and Technological Applications (SRTA-City), 21934 Alexandria, Egypt; eelsayed@srtacity.sci.eg; 2 Composites and Nanomaterials Research Department, Advanced Technology and New Materials Research Institute (ATNMRI), City of Scientific Research and Technological Applications (SRTA-City), 21934 Alexandria, Egypt; melnouby@srtacity.sci.eg; 3 Polymer Materials Research Department, Advanced Technology and New Materials Research Institute, City of Scientific Research and Technological Applications (SRTA-City), 21934 Alexandria, Egypt; mgouda@srtacity.sci.eg; 4 Advanced Technology and New Materials Research Institute (ATNMRI), City of Scientific Research and Technological Applications (SRTA-City), 21934 Alexandria, Egypt; 5 Center of Physics and Engineering of Advanced Materials (CeFEMA), Instituto Superior Técnico, Universidade de Lisboa, 1049-001 Lisbon, Portugal

**Keywords:** cationic dye, photodegradation, nanocomposite hydrogel beads, sodium alginate, tungsten oxide nanostructures, polyvinylpyrrolidone

## Abstract

Tungsten oxide nanostructures were modified by oxygen vacancies through hydrothermal treatment. Both the crystalline structure and morphological appearance were completely changed. Spherical WO_3_·H_2_O was prepared from tungstic acid solution by aging at room temperature, while rod-like WO_3_·0.33H_2_O was prepared by hydrothermal treatment of tungstic acid solution at 120 °C. These structures embedded in sodium alginate (SA)/polyvinylpyrrolidone (PVP) were synthesized as novel porous beads by gelation method into calcium chloride solution. The performance of the prepared materials as photocatalysts is examined for methylene blue (MB) degradation in aqueous solutions. Different operation parameters affecting the dye degradation process, such as light intensity, illumination time, and photocatalyst dosage are investigated. Results revealed that the photocatalytic activity of novel nanocomposite changed with the change in WO_3_ morphology. Namely, the beads with rod nanostructure of WO_3_ have shown better effectiveness in MB removal than the beads containing WO_3_ in spherical form. The maximum degradation efficiency was found to be 98% for WO_3_ nanorods structure embedded beads, while the maximum removal of WO_3_ nanospheres structure embedded beads was 91%. The cycling-ability and reuse results recommend both prepared structures to be used as effective tools for treating MB dye-contaminated wastewaters. The results show that the novel SA/PVP/WO_3_ nanocomposite beads are eco-friendly nanocomposite materials that can be applied as photocatalysts for the degradation of cationic dyes in contaminated water.

## 1. Introduction

Dyes are used broadly in many different industries, including leather, paper-making, cosmetics, tanning, printing, and plastics industries, as well as in the textile and dyeing industries [1,2]. The release of dye-contaminated wastewaters into the environment leads to intensive aquatic defects, which in turn affect the human race. Generally, dyes and their byproducts are considered to be toxic or mutagenic agents [3]. Different strategies are utilized to remove dyes from contaminated waters, such as biological methods [4], coagulation and flocculation [5], photocatalysis [6], and adsorption using natural or synthetic materials [7,8].

Nowadays, degradation by photocatalysis has become a promising technique for the removal of several organic [9] and inorganic [10] pollutants from wastewaters by converting them into non-hazardous substances. Several semiconductor materials have been used as photocatalysts to remove different types of pollutants from wastewaters. One of them is tungsten oxide (WO_3_) [11,12], which has a lower light energy conversion efficiency than the widely used TiO_2_ photocatalyst [13]. WO_3_ has the advantage of being non-toxic, cost-effective, with a high physicochemical stability under irradiation, and a wide range photocatalytic activity in the visible region [14]. It also has good preparation availability of various WO_3_ structures, for instance, orthorhombic WO_3_ has terminal oxygen, which increases its catalytic activity [15]. However, pure WO_3_ has limited photocatalytic activity due to its slow charge transfer and rapid recombination of the photogenerated electron-hole pairs [16]. Several studies have been carried out to tackle this problem. Kim et al. [13] have used thermal evaporation methods to produce WO_3_ nanorods on tungsten substrates. The obtained materials were used for photodegradation of methylene blue (MB) dyes [13]. Jeevitha et al. [17] prepared nanocomposites including both tungsten oxide and graphene oxide as efficient photocatalysts against MB dye. Ma et al. synthesized WO_3_/SnNb_2_O_6_ hybrid nanosheet heterojunctions as efficient Z-scheme photocatalysts via a simple hydrothermal co-assembly method [18]. Ke et al. enhanced the photocatalytic performance of WO_3_ nanosheets for photocatalytic water oxidation by using ion doping (Ag^+^) [19]. Zhang et al. used the co-deposition of noble metals (e.g., Pt) to ameliorate the charge transport between CdS and WO_3_ in CdS/WO_3_ nanojunction [20].

The problem regarding the larger-scale operation of photocatalysis is slow separation and recycling of photocatalyst during the wastewater treatment. This problem can be solved by supporting the photocatalyst onto a polymeric matrix.

Silver embedded in ZnO and polystyrene matrix film was prepared as a floating photocatalyst to remove MB with an efficiency of 97% [21]. ZnO and TiO_2_ nanoparticles were also incorporated into calcium alginate beads as a photocatalyst for the removal of copper ions [10]. A photocatalyst based on TiO_2_ immobilized in calcium alginate beads exhibited an increase in MB degradation efficiency when the beads were reused [22].

A natural polysaccharide polymer, sodium alginate (SA), derived from brown seaweeds, is composed of two acids: α-L-guluronic and β-D-mannuronic acids [23]. SA is biocompatible, non-toxic, biodegradable, gelable polysaccharide, and chelating able, suitable for chemical modification [24] and often used as a polymeric matrix that can act as a catalyst support [10]. SA can be formed as hydrogel beads by cross-linking the α-L-guluronic acid units with poly- or di-valent cations. However, it has some disadvantages as a natural polymer, such as microbial degradation and poor mechanical strength. For improving its usability, it should be blended with synthetic polymer(s) for semi-interpenetrating polymer network hydrogel. Furthermore, the presence of the active functional groups on the natural and synthetic polymers in the polymeric network, allows the obtained hydrogel beads to be used effectively as adsorbents [25].

In this study, polyvinylpyrrolidone (PVP) was blended with SA as a natural pore-former polymer, in order to increase the porosity of the formed beads [26]. In addition, in-situ hydrothermal assembly of WO_3_ nanospheres and nanorods was carried out and the formed nanostructures WO_3_ were embedded with SA/PVP polymer blend to form novel SA/PVP/WO_3_ nanocomposites blend hydrogel beads through a crosslinking method with calcium chloride as the cross-linker. This study aims to (i) prepare a photocatalyst composite by a facile, economic, and efficient method and (ii) investigate the photocatalytic performance of SA/PVP/WO_3_ nanocomposite beads on the removal efficiency of cationic dyes, such as methylene blue. This work focused on doing the performance comparison of WO_3_ photocatalyst with different morphologies and incorporated in porous polymeric beads to form novel hybrid nanocomposites. The obtained nanocomposites were used to remove the organic dye by two mechanisms: Adsorption and photocatalysis.

## 2. Materials and Methods

### 2.1. Materials

Sodium alginate (Sigma Aldrich, St. Louis (MO), USA), polyvinylpyrrolidone (Sigma Aldrich, St. Louis, Missouri, USA), and sodium tungstate hydrate (Na_2_WO_4_·2H_2_O > 99%) was acquired from Kanto Chemicals Co. (Tokyo, Japan). A strong acid type cation-exchange resin (Diaion 25 PK228LH, ion-exchange capacity > 2.05 meq mL^−1^) was purchased from Mitsubishi Chemical Co. (Tokyo, Japan). All chemicals were used without further purification. Deionized water was used for preparing the solutions.

### 2.2. Preparation of WO_3_ Nanostructures

The ion exchange process was done in a glass column with a height of 150 mm and a diameter of 24.6 mm. The glass column was packed with 30 mL of the ion-exchange resin. A flow of 10 mL of water was passed through the column to wash the resin and this washing step was repeated five additional times. 0.5 M of Na_2_WO_4_ solution was prepared by dissolving Na_2_WO_4_·2H_2_O powder in deionized water followed by loading 10 mL of this Na_2_WO_4_ solution on the glass column. The acidified tungstic acid (H_2_WO_4_) solution was recovered by elution with deionized water, with the resulting solution being yellowish and transparent. Inductively coupled plasma atomic emission spectrometry (ICP-AES, Optima 4300DV, Perkin Elmer, Waltham, Massachusetts, USA) was used to determine that the Na^+^ concentration was 1.7 ± 0.6 ppm (n = 3). This ion-exchange precursor was aged at room temperature for 24 h to produce spherical WO_3_ nanoparticles. On the other hand, the ion-exchange precursor was hydrothermally treated at 120 °C for 24 h to produce the WO_3_ nanorods.

### 2.3. Preparation of Adsorbent Beads

Each polymer was dissolved separately in distilled water at room temperature and mixed with percentage containing 90 wt.% SA, 7 wt.% PVP, and 3 wt.% WO_3_ for 2 h to form homogenous solutions. Then, 50 mL of polymer solutions was added dropwise using a syringe into the 200 mL solution containing 2% (*w*/*v*) of calcium chloride. It was allowed to harden for 30 min (during stirring) and then the polymer beads were rinsed three times with distilled water.

### 2.4. Characterization of the WO_3_ Nanostructures and Polymeric Beads

X-ray powder diffraction (XRD, Cu-Kα radiation, Shimadzu-7000, Kyoto, Japan) was used to determine the crystallographic phase of the prepared samples. Their morphology was examined by scanning electron microscopy (SU-70, Hitachi, Tokyo, Japan) combined with energy dispersive X-ray analysis (EDX) for the elements identifications. Fourier transform infrared (FTIR) analysis was done with a Bruker ALFA spectrometer (Bruker Corporation, Ettlingen, Germany).

### 2.5. Photocatalytic Decay Experiments

The evaluation of photocatalytic degradation of MB dye using different prepared SA/PVP/WO_3_ nanocomposite samples, either with WO_3_ nanorods or WO_3_ nanospheres, as a photocatalyst under the illumination of unfiltered and commercially available LED visible light was carried out using a Plexiglas cylindrical reactor with 15 cm diameter and 20 cm height, as shown in Figure S1 (Supplementary Information). The glass surface of the reactor was covered with aluminum foil. In addition, the reactor had two 12 W lamps with a light intensity of 1200 lm (Bareeq, Cairo, Egypt) fixed on the top as the radiation source.

Typically, 0.5 g L^−1^ of the catalyst was suspended in a model wastewater of MB dye solution. The suspension was magnetically stirred at room temperature and illuminated with visible light, with the samples being collected at a regular interval of time. The residual MB concentration after irradiation was monitored using UV–Vis spectrophotometer (Shimadzu UV-2600, Kyoto, Japan) at 665 nm by sampling 2 mL of the reaction mixture. The photocatalytic degradation of MB using different WO_3_ morphologies was calculated using the formulae,

photodegradation % = [(C_0_ − C)/C_0_] × 100 (1)

where C_0_ and C are the initial and final dye concentrations, respectively.

The photocatalytic efficiency of the two different prepared composites beads on MB was studied at pH 7 of the dye solution. The pH was adjusted to seven by adding 0.1 M HCl or 0.1 M NaOH. Habib et al. [27] have reported that a pH value of seven is the most suitable for photocatalytic degradation activity.

### 2.6. Kinetic Models

The pseudo-first-order model and the pseudo-second-order model are the most common kinetic models used. The pseudo-first-order equation was stated as follows [28],

(2) $$\log(q_{e}-q_{t})=logq_{e}-K_{1}t$$

where *q_e_* and *q_t_* are the amounts of MB adsorbed or degraded (mg g^−1^) at equilibrium and at time *t* (sec), respectively, and *k*_1_ is the rate constant (sec^−1^). Values of *K*_1_ were calculated from the plots of log (*q_e_* − *q_t_*) versus *t*. The pseudo-second-order model can be expressed by the following expression [8],

(3) $$\frac{t}{q_{t}}=\frac{1}{K_{2}q_{e}{}^{2}}+(\frac{1}{q_{e}})t$$

where *K*_2_ (g mg^−1^ s^−1^) is the rate constant of the second-order model. The plot of *t*/*q* versus *t* should show a linear relationship when the second-order kinetics is applicable. *q_e_* and *K*_2_ can be determined from the slope and intercept of the plot. This procedure is probably predicting the behavior over the whole range of processes.

## 3. Results and Discussion

### 3.1. Characterization of the SA/PVP/WO_3_ Nanocomposite

**Figure 1:**
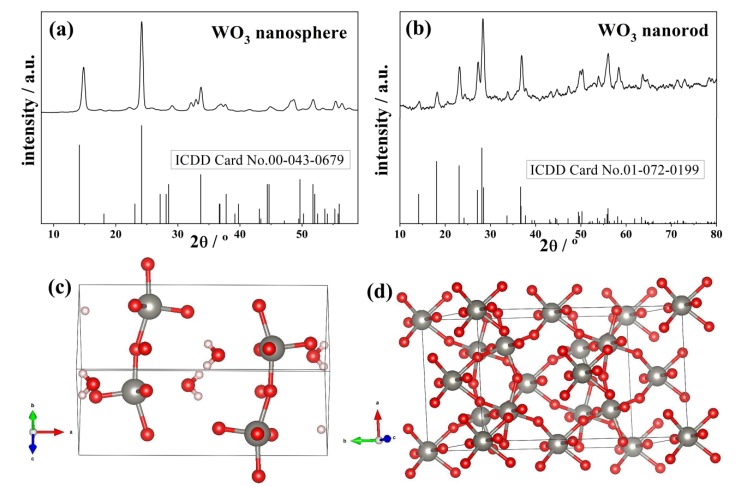
XRD patterns of (**a**) the WO_3_ nanospheres (ICDD Card No. 00-043-0679) and (**b**) WO_3_ nanorods (ICDD Card No. 01-072-0199) and 3D chemical structure for the (**c**) WO_3_ nanospheres and (**d**) WO_3_ nanorods.

The crystal structure of the synthesized WO_3_ nanospheres and nanorods was checked by XRD analysis. The XRD patterns of both WO_3_ nanoparticles show highly crystalline features and it is close to the literature data of the orthorhombic WO_3_·H_2_O phase [29,30]. The WO_3_ nanosphere was in orthorhombic WO_3_·H_2_O phase with space group of *Pmnb* (62) and lattice parameters of a = 5.2380 Å, b = 10.7040 Å, and c = 5.1200 Å (ICDD Card No. 00-043-0679) (Figure 1a). On the other hand, the WO_3_ nanorods were in orthorhombic WO_3_·0.33H_2_O phase, with a space group of *Fmm2* (42) and lattice parameters of a = 7.3590 Å, b =12.75130 Å, and c = 7.7040 Å (ICDD Card No. 01-072-0199) (Figure 1b). For both crystals, there was no secondary phase detected, although the WO_3_·0.33H_2_O nanorods contain more oxygen vacancies than WO_3_·H_2_O nanospheres, as shown in Figure 1c,d.

**Figure 2:**
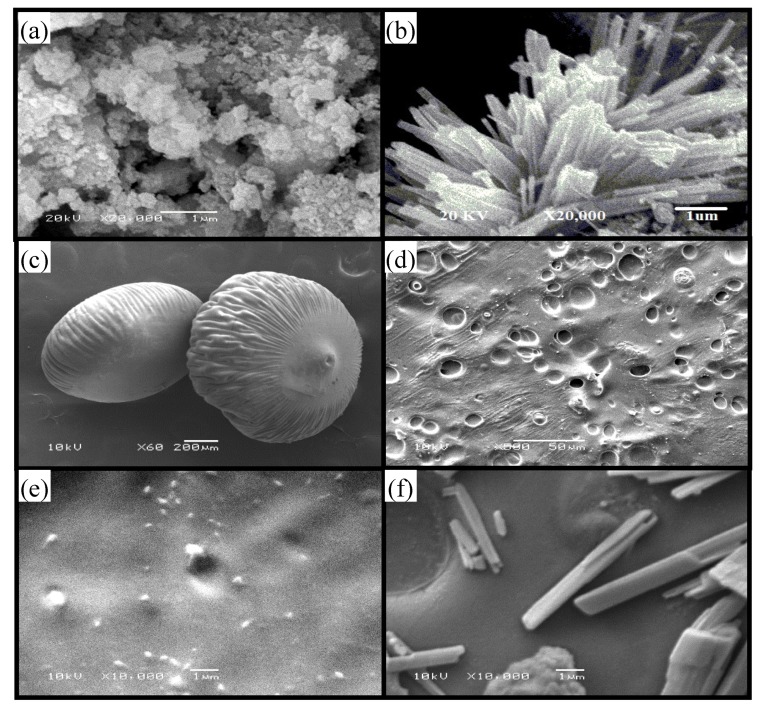
SEM images of (**a**) the WO_3_ nanospheres, (**b**) the WO_3_ nanorods, (**c**) spherical-shaped beads, and high magnification of (**d**) the bead surface and the inside of (**e**) the nanosphere and (**f**) the nanorod nanocomposite beads.

The SEM images in Figure 2a,b show the morphology of the synthesized WO_3_ nanoparticles as agglomerated or individual nanospheres and nanorods, respectively. It is noticeable from the presented XRD and SEM results that both the morphology and the crystalline structure agree with previous researches [30,31]. After embedding the WO_3_ nanoparticles into the blended SA/PVP polymers to obtain SA/PVP/WO_3_ nanocomposite beads, the SEM images of the beads were found to be rough and wrinkled (Figure 2c) with visible pores (Figure 2d). Meanwhile, by taking a high magnification inside the bead, a good dispersion of the WO_3_ nanospheres or nanorods was observed (Figure 2e,f, respectively).

**Figure 3:**
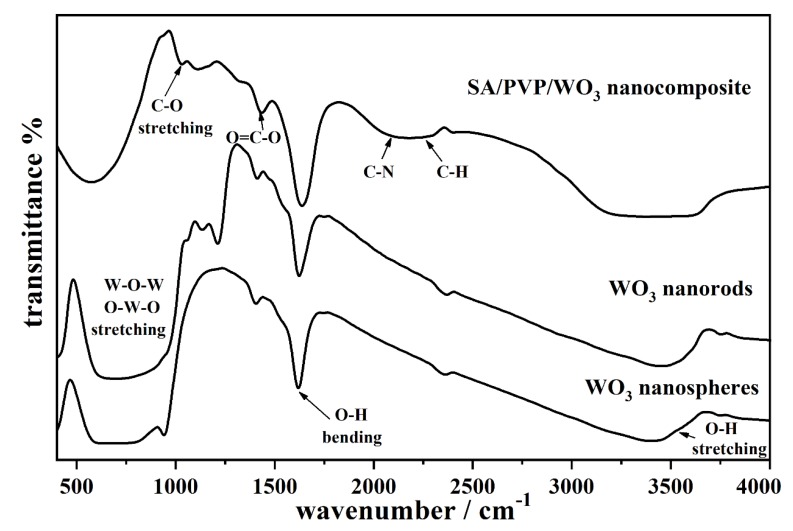
FTIR spectra of the WO_3_ nanospheres, WO_3_ nanorods, and sodium alginate (SA)/polyvinylpyrrolidone (PVP)/WO_3_ nanocomposite.

Figure 3 shows the FTIR spectra of WO_3_ nanospheres, WO_3_ nanorods, and SA/PVP/WO_3_ nanocomposite. The FTIR measurements for WO_3_ nanosphere and WO_3_ nanorod reveal, in general, that their patterns are composed of a bond between W and O, and in particular that the band in the 500–1000 cm^−1^ range is characteristic of the W-O-W and O-W-O stretching vibration modes [32]. Moreover, all samples show bands around 1600 and 3500 cm^−1^ that are attributed to the O-H stretching bending modes and the H_2_O bending vibration modes, respectively. On the other hand, the FTIR spectrum for SA/PVP/WO_3_ nanocomposite shows absorption bands at 1419 cm^−1^, which is characteristic of symmetric stretching vibration of (COO) groups for SA. The band at 1030 cm^−1^ represents skeletal stretching of (C–O) [33], the band at 2178 cm^−1^ corresponds to (C-N) bond of PVP, and the band at 2170–2300 cm^−1^ is due to the (C-H) bonds of the polymers [26]. The WO_3_ vibrations were found at the 600–1000 cm^−1^ region.

### 3.2. Adsorption and Photocatalysis Removal of Methylene Blue

#### 3.2.1. Effect of Time on MB Decay

**Figure 4:**
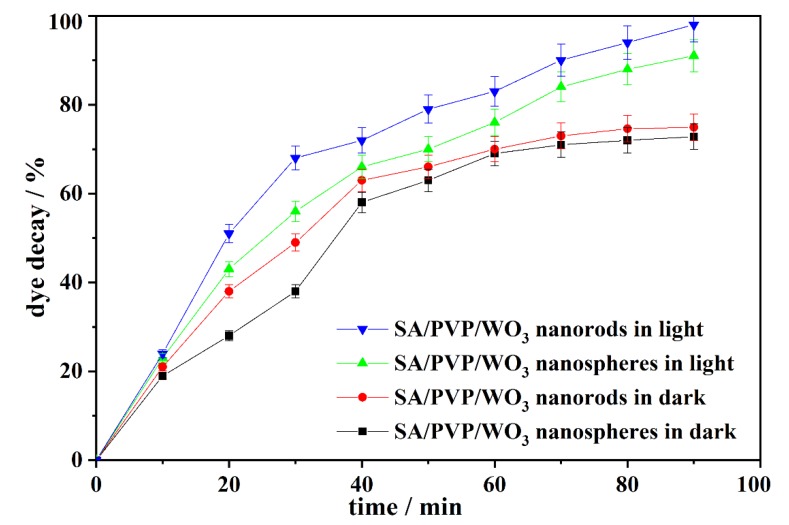
The relation between illumination time and methylene blue (MB) decay (%) using the SA/PVP/WO_3_ nanorods and the SA/PVP/WO_3_ nanospheres nanocomposites.

The adsorption behavior of SA/PVP/WO_3_ nanocomposites was studied in the dark to determine the adsorption extent for MB dye onto the beads. That information is required to assess the photocatalytic activity of the SA/PVP/WO_3_ nanocomposite beads for removing MB dye in the presence of light. The illumination time was performed using 0.5 g L^−1^ of the two different composite beads with 500 mL of 50 mg L^−1^ dye solution at solution pH equal to seven, in dark and under visible light at different time intervals. As shown in Figure 4 and illustrated in Table S1 in the Supplementary Information, dark adsorption increased with the increasing time, and then almost plateaued after 60 min, which may be due to the porous nature of the SA/PVP blended polymer. Additionally, the most abundant functional groups in SA polymer are carboxylic groups, which also enhance the adsorption of cationic dye molecules.

However, under visible light, the results reveal that there was an increase in the photocatalytic activity with the increase in illumination time reaching 98% after 90 min of illumination using SA/PVP/WO_3_ nanorods composite and 91% after 90 min using SA/PVP/WO_3_ nanospheres composite.

#### 3.2.2. Effect of the Light Intensity on the MB Photocatalytic Decay

**Figure 5:**
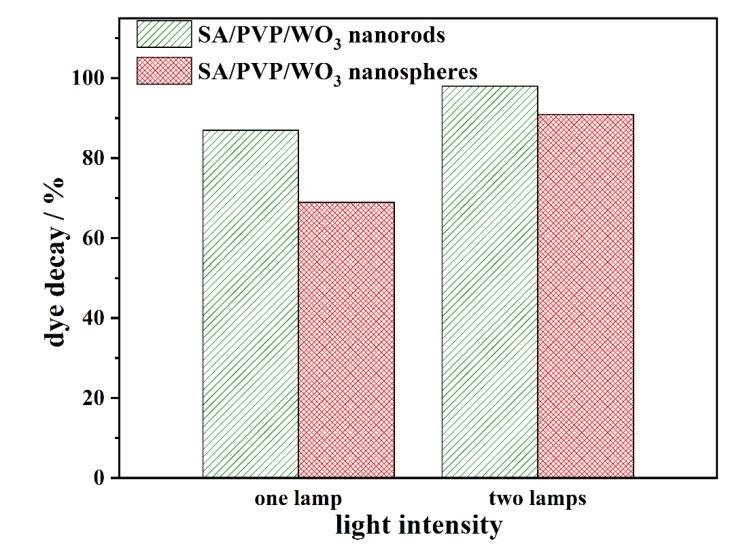
Effect of light intensity on the photocatalytic MB decay using SA/PVP/WO_3_ nanocomposites (pH 7; 90 min of illumination time; and 50 mg L^−1^ as the initial MB concentration).

The effect of different light intensities on the effectiveness of photocatalytic dye decay using the composites with the two different morphologies of WO_3_ was assessed. The effect of UV light intensity on the efficiency of the system was evaluated by fixing two lamps on the reactor cover instead of one lamp. The results presented in Figure 5 indicate that increasing the light intensity increased the efficiency of the system after 90 min. This is because the increase of the light intensity increases the quantity of light received by the photocatalyst particles, which increases electron stimulation and enhances the system’s effectiveness [34].

#### 3.2.3. Effect of Initial Dye Concentration on MB Photocatalytic Decay

**Figure 6:**
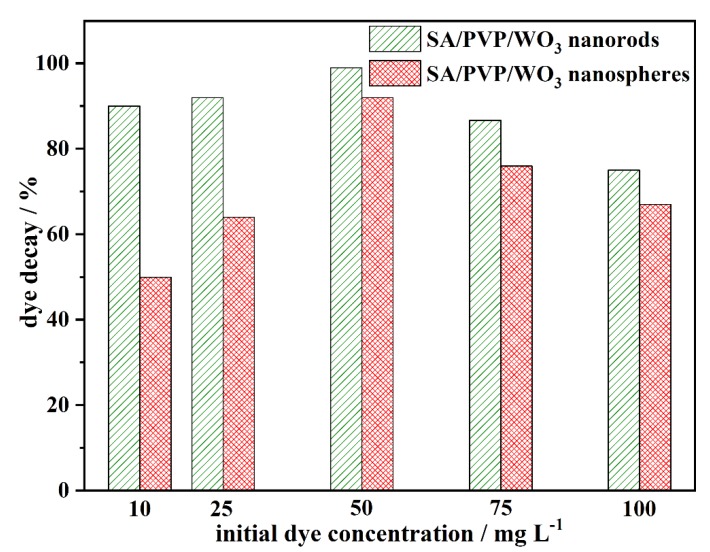
Effect of initial MB dye concentration on photocatalytic decay process using the two prepared nanocomposite photocatalysts (pH 7; 90 min contact time; and 1200 lm of light intensity).

Figure 6 shows the effect of initial dye concentration on the MB decay using the two prepared photocatalysts. In a typical process, 0.5 g L^−1^ (fixed) of catalysts were added to the different solutions of dye with different concentration (10, 25, 50, and 100 mg L^−1^) maintaining the dye solution at pH 7 with a contact time of 90 min and light intensity of 1200 lm. However, the results reveal that the MB dye decay generally increases with the increasing initial MB concentration, with the maximum decay being obtained at 50 mg L^−1^. Further increase in the MB dye concentration has a negative effect on its decay.

This can be returned to the fact that dyes photodegradation rate depends mainly on the possibility of formation of hydroxyl free radicals OH^•^ on the catalyst surface and the reaction of the dye molecules with the formed radicals. The initial increase in the decay rate with the increase in initial dye concentration might be attributed to an increase in the probability of the reaction between the dye molecules and OH^•^ [35,36]. The proposed mechanism for MB degradation can be summarized in the following steps. First, visible light irradiation allows electrons in the valence band to transfer into the conduction band. Hence, holes (h) and electrons (e−) are formed at the surface of the WO_3_ photocatalyst. Then, the holes react with hydroxide ion, while electrons react with dissolved oxygen for the production of OH^•^, which degrade MB dye into non-toxic gases, such as carbon dioxide, and water. Furthermore, hydrogen peroxide reacts with electrons for the production of more OH^•^ for enhancing the degradation of the dye [37,38,39].

WO_3_ + visible light → h^+^ (hole) + e^−^ (electron) (4)

h^+^ + H_2_O → H^+^ + OH^−^ (5)

h^+^ + OH^−^ →OH^•^ (6)

e^−^ + O_2_ → O_2_^•−^ (7)

O_2_^•−^ + e^−^ + 2H^+^ → H_2_O_2_ (8)

O_2_^•−^ + H_2_O_2_ → OH^•^ + OH^−^ + O_2_ (9)

OH^•^ + dye → nontoxic products + CO_2_↑ (10)

O_2_^•−^ + dye → nontoxic products + CO_2_↑ (11)

However, a further rise in the MB concentration decreases the activity of the photocatalyst. This might be due to the inhibition of the reaction between the MB dye molecules and OH^•^, as more MB molecules are adsorbed on the catalyst surface at high dye concentration thereby reducing the formation of OH^•^ [40]. Moreover, with increased color intensity, gaps pertaining to the entry of photons get restricted from reaching the surface of the photocatalyst limiting the radical attack (photodegradation) of pollutants [39]. Hence, initial MB concentration was found to be optimum at 50 mg L^−1^ exhibiting 98% photodegradation efficiency using SA/PVP/WO_3_ nanorods nanocomposite.

#### 3.2.4. Effect of WO_3_ concentration in SA/PVP matrix

**Figure 7:**
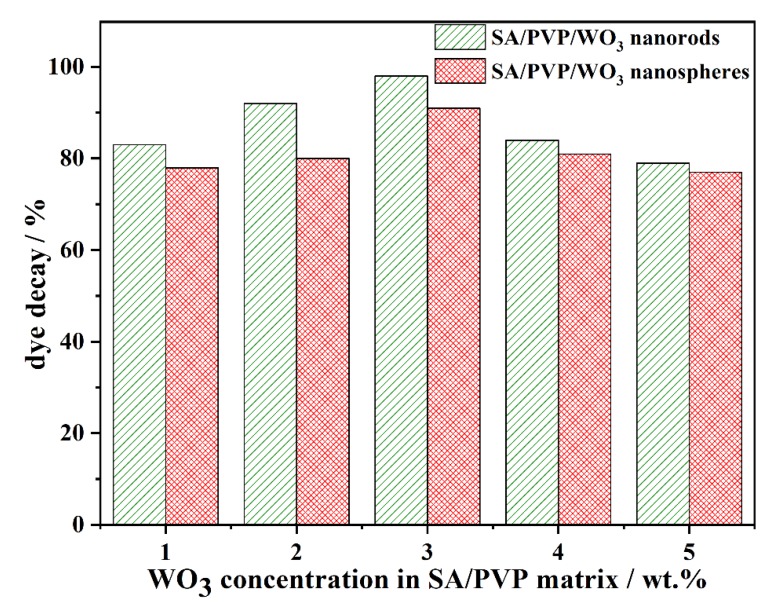
Effect of catalyst loading on the dye decay (pH 7; 90 min illumination time; 50 mg L^−1^ initial MB concentration; and 1200 lm of light intensity).

The effect of photocatalyst concentration on MB decay was studied by varying the prepared WO_3_ photocatalysts concentration in SA/PVP matrix between 1 to 5 wt.%. From Figure 7 it can be concluded that, with increasing catalyst load from 1 to 3 wt.%, the adsorption efficiency of the system decreases and photodegradation efficiency increases. This may be justified by the fact that at very low catalyst load more porous vacant sites are available on the outer surface of beads to absorb the dye molecules, in addition to the polymers functional groups (COO^−^), which react with the cationic dyes by electrostatic attraction but the available active sites for the photocatalytic reaction are not sufficient. On the other hand, by increasing the catalyst load to 3 wt.% more active sites for the photocatalytic reaction are available providing more chances for the hydroxyl ions adsorption onto the surface producing superoxide radicals. Meanwhile, at higher catalyst loading the photocatalytic activity was reduced, with the further increment in catalyst loading hampering the dye decay rate due to a shortage of light penetration [41]. Though both forms of WO_3_ have terminal oxygen, the highest photocatalytic activity of WO_3_·0.33H_2_O nanorod may be due to higher oxygen vacancies. Y. Li et al. reported that the more oxygen vacancies WO_3_ crystal has, the higher the photocatalytic activity will be [42].

However, the catalyst load in SA/PVP/WO_3_ nanocomposite played a major role in the removal efficiency of dye contaminants. In other words, it will define that either adsorption or photocatalysis will dominate on the removal process, and it should be optimized to achieve better removal from the system and allow being reused several times without losing catalytic activity in the photocatalytic dye removal.

### 3.3. Analysis of the Reaction Kinetics and Proposed Mechanism

#### 3.3.1. Kinetic Models

Pseudo-first-order and pseudo-second-order kinetic models are applied for the investigation of the reaction mechanism of SA/PVP/WO_3_ nanocomposite with MB. Generally, kinetic models describe the reaction rates while the order for the reaction defines the dependence of the reaction rates on the concentrations of reacting species [43]. Herein, two sets of experiments were done, in the dark and under light irradiation. Depending on the obtained results, as illustrated in Table 1, it was noticed that there are differences between the two kinetic models and their correlation coefficient (R^2^) values in the dark and in light irradiation. In dark mode, the rate constants K_2_ value of the pseudo-second-order model is high for SA/PVP/WO_3_ rod-like, which indicates the chemisorption nature for the adsorption process of MB [44]. Under light irradiation, the R^2^ value of the pseudo-first and second-orders model are the same for the rod-like WO_3_, which may be due to the high chemical stability of the prepared catalyst under light irradiation conditions.

#### 3.3.2. Proposed Reaction Mechanism of SA/PVP/WO_3_ Nanocomposite with MB

In general, the orthorhombic structure of WO_3_ contains terminal oxygen atoms as “unsteady state atoms”, and at pH region of three to seven, the surface of WO_3_ has a negative charge. Consequently, these “unsteady state” oxygen atoms interact with nitrogen atoms in MB molecules exhibiting faster adsorption property to MB [15]. Under light irradiation, electron-hole pairs are formed and concentrated on the surface [45]. On the oxygenated media, OH^−^ and O_2_^−^ radicals are produced (Figure S2 in Supplementary Materials). Finally, these superoxide radicals ion or the hydroxyl radicals degrade the MB dye into small molecular fragments, e.g., CO_2_, H_2_O, and H^+^, as the final products (Equations (4)–(11)). In summary, the degradation mechanism starts with the MB dye adsorption on the nanocomposite surface followed by its photodegradation [46].

The herein prepared photocatalysts are compared in Table 2 with other WO_3_-based nanocomposites previously tested for the removal of different organic dyes from water. The removal efficiency of tungsten oxide nanorod embedded in sodium alginate/polyvinylpyrrolidone composite was higher than that of the tungsten oxide-based counterpart. The comparison is very clear between the prepared composite and the aligned WO_3_ [47], tungsten-loaded TiO_2_ [48], and mesoporous WO_3_/TiO_2_ [49], where the removal efficiency percentages are up to 98%, up to 94%, 90%, and up to 88%, respectively. Although the removal efficiency is closer to that of WO_3_-graphene oxide (WO_3_-GO) [17] and WO_3_ nanorods on reduced graphene oxide [11], the cost and methodology of preparation of these materials is unfavorable when compared to the herein prepared nanocomposite beads, which recommends its practical application.

### 3.4. Recycling of SA/PVP/WO_3_ Nanocomposite

**Figure 8:**
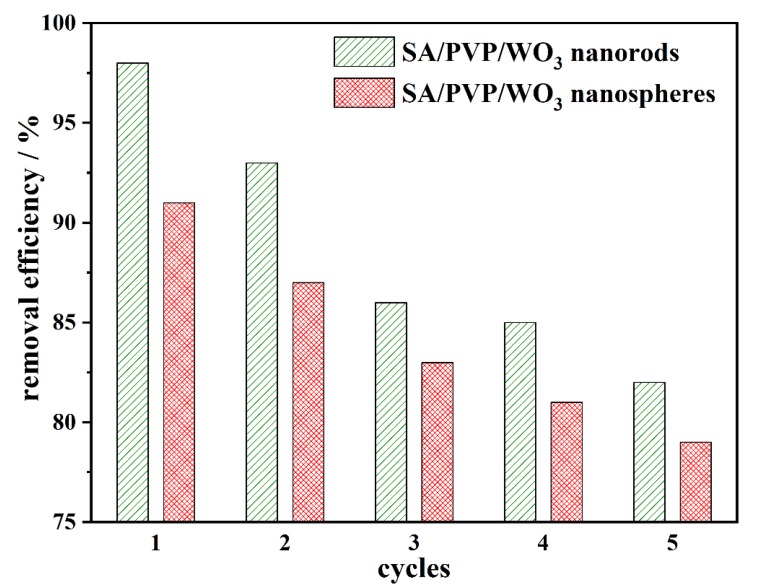
The recycling efficiency of SA/PVP/WO_3_ nanocomposites during MB removal for 5 consecutive cycles (pH 7; 90 min illumination time; and 50 mg L^−1^ initial MB concentration).

To study the reusability of SA/PVP/WO_3_ nanocomposite, which allows the process to be regarded as cost-effective, five experimental runs were carried out at optimized conditions using the same beads and degradation efficiency for MB (Figure 8). The SA/PVP/WO_3_ nanocomposites were recovered and washed with 0.1 M HCl solution and used for five times. The obtained results show that the efficiency decreases from 98% to 82% for used WO_3_ nanorods and decreases from 91% to 79% for used WO_3_ nanospheres. This may be attributed to the fouling of the porous surface of the composite [9].

## 4. Conclusions

Tungsten oxide with two different morphologies was embedded in sodium alginate/ polyvinylpyrrolidone as blended polymers. The prepared SA/PVP/WO_3_ nanocomposite beads were employed for the removal of methylene blue dye in aqueous solutions under visible light. From the obtained MB removal profiles, SA/PVP/WO_3_ nanorods composite beads have performed better than SA/PVP/WO_3_ nanospheres composite beads. The removal of MB dye is not solely driven by the adsorption capability of the nanocomposite beads, but it is also attributed to the photocatalytic properties of the WO_3_. The mechanisms of MB dye removal can be explained in the following steps. The initial adsorption of MB dye molecules onto the SA/PVP/WO_3_ beads is followed by the photocatalytic degradation of the adsorbed dye molecules by WO_3_ nanoparticles. Thus, adsorption and photocatalysis are proposed as the main steps in the removal of MB dye and the composite beads can remove the cationic dye molecules by using the concept of “absorb and degrade”. From this study, it is demonstrated that the prepared composite beads can be easily recovered and reused as effective tools for treating MB dye-contaminated wastewaters.

## Figures and Tables

**Table 1:** Pseudo-first order and pseudo-second-order kinetic parameters.

Nanocomposite Material	Pseudo-First-Order			Pseudo-Second-Order	
	q_e_ mg g^−1^	K_1_ sec^−1^	R^2^	K_2_ g mg^−1^ s^−1^	R^2^
**SA/PVP/WO_3_ Sphere Nanocomposite in Dark**	72.8	0.057	0.954	0.000398238	0.902
**SA/PVP/WO_3_ Rods Nanocomposite in Dark**	74.9	0.062	0.920	0.000533691	0.979
**SA/PVP/WO_3_ Sphere Nanocomposite in Light**	91.0	0.039	0.948	0.000358546	0.992
**SA/PVP/WO_3_ Rods Nanocomposite in Light**	98.0	0.037	0.973	0.000368839	0.973

**Table 2:** Tungsten oxide based nanocomposites and its photodegradation behavior against organic dyes.

Materials	Structure	Morphology	Dye	Efficiency	Ref.
tungsten oxide embedded in sodium alginate/polyvinylpyrrolidone composite beads	orthorhombic crystalline WO_3_	spherical, nanorods	MB	up to 98%	current work
aligned WO_3_	triclinic, orthorhombic and monoclinic	nanorods and nanosheets	MB	up to 94%	[47]
tungsten-loaded TiO_2_	crystalline WO_3_ at higher loadings (>12 mol%)	aggregation	MB	90%	[48]
mesoporous WO_3_/TiO_2_	crystalline	mesoporous	rhodamine B	up to 88%	[49]
MWCNT/WO_3_	hexagonal and orthorhombic	aggregation	rhodamine B	up to 92%	[50]
α-Fe_2_O_3_/WO_3_ composite	crystalline	spherical-shaped α-Fe_2_O_3_ nanoparticles and WO_3_ nanorods	MB	up to 91%	[51]
tungsten oxide-graphene oxide (WO_3_-GO)	monoclinic	aggregation	MB	97%	[17]
WO_3_ nanorods on reduced graphene oxide sheets	hexagonal wurtzite phase	flower-like	methylthionine chloride	94%	[11]

## Supplementary Materials

The following are available online at https://www.mdpi.com/1996-1944/13/8/1905/s1, Figure S1: The used photocatalytic reactor, Figure S2: (**a**) Schematic illustration of the interaction between “unsteady state” oxygen atoms on WO_3_ and nitrogen atoms in MB molecules and (**b**) the suggested MB photodegradation mechanism, Table S1: Average experimental values* of the relation between illumination time and MB degradation (%) using the SA/PVP/WO_3_ nanorods and SA/PVP/WO_3_ nanospheres nanocomposites.
