# Supplementary material for: Effect of the Morphology of Tungsten Oxide Embedded in Sodium Alginate/Polyvinylpyrrolidone Composite Beads on the Photocatalytic Degradation of Methylene Blue Dye Solution
Source: Materials (Basel). 2020 Apr 17;13(8):1905. doi: 10.3390/ma13081905 (PMC7216279; doi:10.3390/ma13081905)
Supplement: Supplementary file 1 [file materials-13-01905-s001.pdf]

materials

Supplementary Material

Effect of the Morphology of Tungsten Oxide Embedded in Sodium Alginate/Polyvinylpyrrolidone Composite Beads on the Photocatalytic Degradation of Methylene Blue Dye Solution

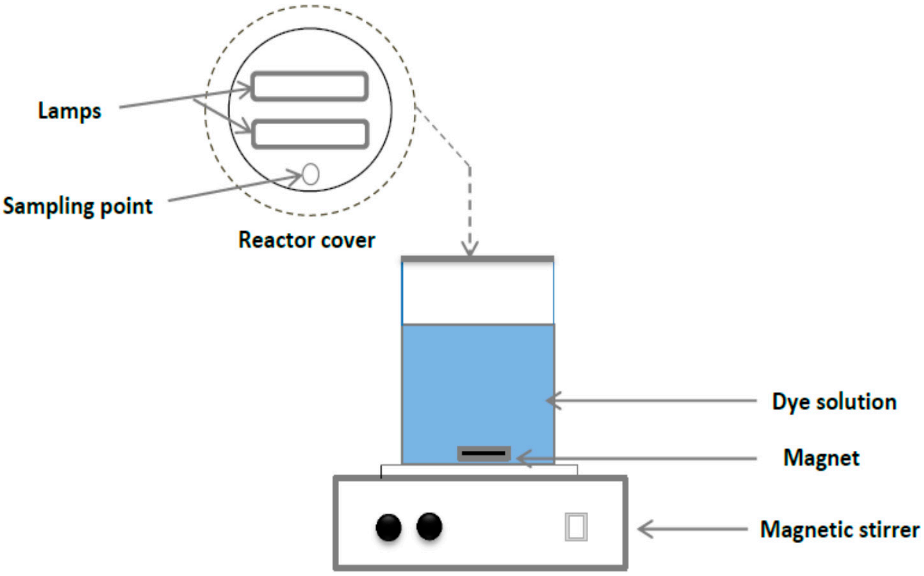

Figure S1. The used photocatalytic reactor.

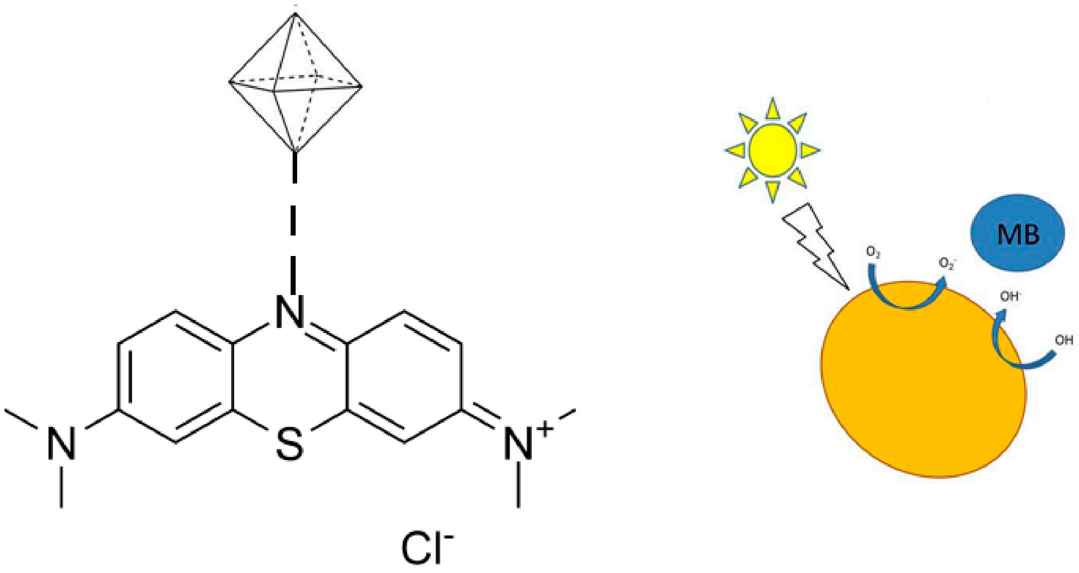

(**a**) (**b**)

Figure S2. (**a**) Schematic illustration of the interaction between "unsteady state" oxygen atoms on WO_3 and nitrogen atoms in MB molecules and (**b**) the suggested MB photodegradation mechanism.

Table S1. Average experimental values* of the relation between illumination time and MB degradation (%) using the SA/PVP/WO_3 nanorods and SA/PVP/WO_3 nanospheres nanocomposites.

Time (min.)	Dye Removal % with SA/PVP/WO_3 Nanospheres in Dark	Dye Removal % with SA/PVP/WO_3 Nanorods in Dark	Dye Removal % with SA/PVP/WO_3 Nanospheres in Light	Dye Removal % with SA/PVP/WO_3 Nanorods in Light
10	19 ± 0.2	21 ± 0.1	23 ± 1.2	24 ± 0.8
20	28 ± 0.2	38 ± 0.3	43 ± 1.3	51 ± 0.9
30	38 ± 0.4	49 ± 0.3	56 ± 1.4	68 ± 0.8
40	58 ± 0.7	63 ± 0.5	66 ± 0.6	72 ± 1.2
50	63 ± 0.3	66 ± 0.4	70 ± 1.0	79 ± 1.1
60	69 ± 0.5	70 ± 0.5	76 ± 0.9	83 ± 1.0
70	71 ± 0.4	73 ± 0.5	84 ± 1.4	90 ± 1.1
80	72 ± 0.2	74.6 ± 0.8	88 ± 1.2	94 ± 0.9
90	72.8 ± 0.5	74.9 ± 0.6	91 ± 1.0	98 ± 1.3

*average values obtained from 3 replicates
